# Application of blended learning approach in clinical skills to stimulate active learning attitudes and improve clinical practice among medical students

**DOI:** 10.7717/peerj.11690

**Published:** 2021-06-24

**Authors:** Jie Gong, Manzhen Ruan, Wen Yang, Miao Peng, Zhen Wang, Lichen Ouyang, Guangyao Yang

**Affiliations:** 1 The Clinical Skill Center, The First Clinical College, Union Hospital, Tongji Medical College, Huazhong University of Science and Technology, Wuhan, China; 2 Department of Immunology, School of Medicine, Jianghan University, Wuhan, China

**Keywords:** Medical students, Blended learning, Clinical skills, Active learning

## Abstract

**Background:**

The recent application of blended educational methods has impacted medical education and has drawn attention to a new teaching method. This teaching style presents unique opportunities and challenges. We investigated the effects of blended learning and traditional teaching methods on clinical skill development.

**Methods:**

We sorted 200 medical students from Tongji Medical College at Huazhong University of Science and Technology into a control or experimental group. The control group was taught with a traditional lecture-based learning method and the experimental group was taught using a blended learning method. The two groups were compared after training to assess their theoretical and practical differences. A student satisfaction survey was given to participants in both groups.

**Results:**

The results of the experimental group’s theoretical and practical assessments were found to be significantly higher (*p* < 0.05) than that of the control group. The student satisfaction survey showed that blended learning was significantly more effective for acquiring relevant knowledge, enhancing student-centered learning and improving clinical practice.

**Conclusions:**

Blended learning may address deficiencies in clinical skills, make up for limited time and space, and ensure learning efficiency and quality.

## Introduction

The application of modern technology to medical education in China has led to new methods of teaching. However, clinical skills are still taught by traditional lecture-based learning (LBL) and evidence suggests that students receive more theoretical knowledge than hands-on learning. Clinical skills are fundamental to the practice of medicine and form a core component of physicians’ professional identity (Elder, 2018). Clinical skills curricula are needed to connect medical knowledge with practical and operational skills. Physicians’ clinical competencies are closely related to the life and safety of their patients. Clinical skills must be taught and practiced to ensure that medical students master skills, practice proficiency, and avoid medical errors to protect their patients’ lives, safety, and rights. Therefore, it is important to determine how best to improve teaching techniques and enhance students’ practical skills.

Blended learning refers to the systematic integration of online and face-to-face learning in order to facilitate critical, creative, and complex thinking skills (Garrison & Kanuka, 2004). In this process, students study before class and then apply their knowledge in classroom discussion and through practical operation (Westerlaken et al., 2019). Unlike the passive teaching in the traditional in-class lectures, blended learning shifts the focus of education from teacher-centered to student-centered. It also has the potential to increase student-teacher interaction, leading to improved learning efficiency (Hake, 1998). Studies have revealed that blended learning may increase education levels and stimulate effective learning for postgraduate health- and dental-care professionals (Westerlaken et al., 2019; Varthis & Anderson, 2018). Furthermore, blended learning reportedly achieved better student outcomes than traditional face-to-face teaching in gross anatomy courses (Green et al., 2018). Another study has shown that traditional teaching methods were increasingly unable to fulfill medical students’ needs or complement their learning habits (Cheng et al., 2017).

Blended learning has benefited from technological developments, such as virtual reality and screen-based simulation (Dyer, Swartzlander & Gugliucci, 2018; Eagleton, 2017; Kannan & Kurup, 2012). The rapid development of computer network technology has been widely used in various fields of medical education and has significantly transformed how we conceptualize education (Izard et al., 2018). A screen-based simulation is a form of computer-generated technology that creates a multifaceted virtual environment for the observer (Mahmood et al., 2018). Screen-based simulations are used in a variety of applications (e.g., simulate anesthetic procedures) to improve anatomic conceptualization and enhance clinical performance (Schwid et al., 2001; Nyssen et al., 2002). Screen-based simulations are being applied with greater frequency in clinical medicine to aid with pain management, rehabilitation, resuscitation for cardiac arrest, and general clinical education (Bonnetain et al., 2010; Ali et al., 2017).

Several studies have reported on students’ perceptions of active learning strategies in various disciplines (Hurtubise et al., 2015; Sharma et al., 2015). However, few studies have analyzed the use of the blended learning approach in clinical skills. The First Clinical College of the Tongji Medical College of Huazhong University of Science and Technology used blended learning as an option for teaching clinical skills due to the rapidly increasing volume of materials available online. We studied the blended learning approach in a clinical skills curriculum and quantitatively compared the performance of those taught using a blended learning approach versus a traditional teaching method.

### Methods

### Participants

The clinical skills curricula is normally offered to the clinical medical undergraduates at the Tongji Medical College of Huazhong University of Science and Technology in their fourth year as they begin clinical practice. One hundred students voluntarily participated in our pilot blended learning program. Written informed consent was obtained from all participants in the experimental group prior to the study. A total of 100 non-participating students were followed and designated as the control group. These students were taught using traditional teaching methods to evaluate the efficacy of the blended learning approach. We obtained approval for this study from the Medical Research and Ethics Committee, Union Hospital, Tongji Medical College, Huazhong University of Science and Technology [Ethical Application Ref: [2020] IEC-J (017)]. There were no statistically significant differences between the control group and experimental group in terms of age, gender, and examination scores in the third year of medical college (Table 1).

**Table 1:** Demographic characteristics of the blended learning participants and control group.

Group	Gender	Age	Examination scores in medical school
Experimental group (*n* = 100)	Male: n = 50 Female: n = 50	22.00 ± 0.35	85.57 ± 3.73
Control group (*n* = 100)	Male: n = 51 Female: n = 49	21.99 ± 0.39	84.54 ± 4.11
*t*		0.192	1.864
*p*		0.848	0.064

Medical students in the control group and the experimental group were taught using the traditional LBL method and blended learning in other curricula. The clinical skills curricula was based on the training objectives of the National Clinical Medicine training plan and the requirements of the National Medical Practitioner Examination. As illustrated in Table 2, the curricula included common clinical skills such as emergency medicine, internal medicine, surgery, gynecology, and pediatrics. The curriculum in the control and experimental group consisted of bi-weekly theoretical sessions (2 h in the classroom) and bi-weekly experimental practical sessions (3-h sessions to practice techniques).

**Table 2:** The curriculum arrangement of clinical skills.

The contents of curriculum	Theoretical period (hours)	Experimental period (hours)
Cardiopulmonary resuscitation; trachael intubation	2	3
Pediatric physical measurement; neonatal resuscitation	2	3
Techniques of trauma first aid	2	3
Arterial and venous puncture; aspiration of sputum	2	3
Dressing change and suture removal; gastric tube insertion	2	3
Thoracentesis; abdominocentesis	2	3
Bone marrow puncture; lumbar puncture	2	3
Pelvic examination, Four maneuvers of leopold	2	3
Urethral catheterization	2	3

**Notes.**

The curriculum in the control and experimental group consists of bi-weekly theoretical sessions (2-h long in the classroom) and bi-weekly experimental practical sessions (3-h sessions for practice the skill techniques).

### Intervention methods in the control group

A traditional teaching method consisting of in-class lectures and simulation-based clinical training was applied in the control group. In traditional in-class lectures, teachers introduced theoretical knowledge of clinical skills in the form of lectures and demonstrated techniques for students. In the simulation-based clinical training, a small class of eight to ten students per group practiced the different clinical skills, such as thoracentesis, lumbar puncture, and marrow aspiration, on normalized simulators.

### Intervention methods in the experimental group

**Figure 1:**
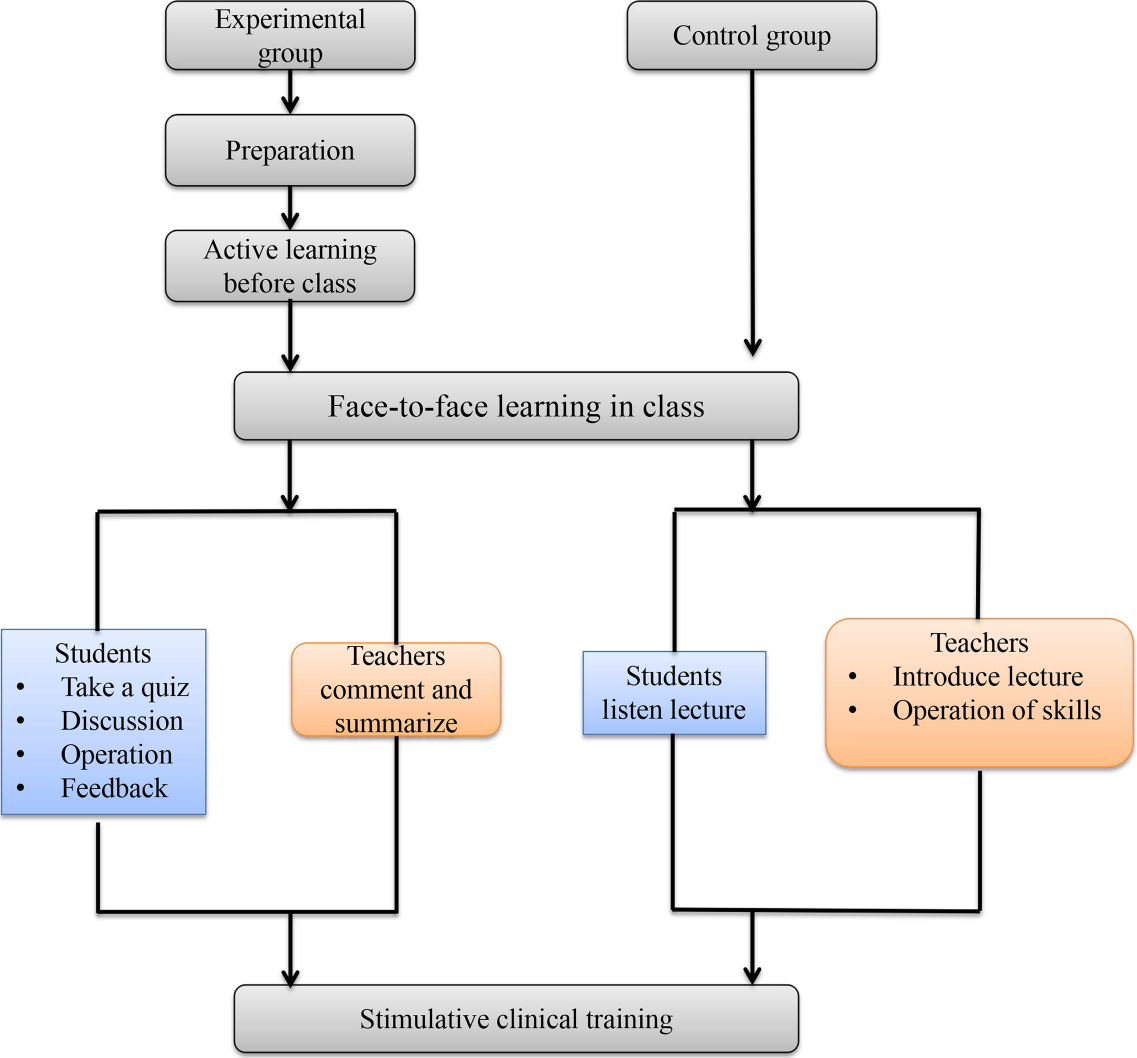
Schematic diagram demonstrating the difference of the blended learning and traditional teaching models.

We created a website with several online platforms including micro-lectures, demonstration videos, online exercises, screen-based simulation of clinical skills, and a student-teacher communication platform in order to achieve efficient, independent learning. As shown in Fig. 1, blended learning was divided into four parts: preparation, active learning before class, face-to-face learning in class, and simulative clinical training.

1. ***Preparation:*** Teachers prepared and uploaded didactic content to the First Clinical College’s website two weeks prior to class. The online resources included micro-lectures, demonstration videos of clinical skills, exercises, and screen-based simulation of clinical skills.
2. ***Active learning before class*:** In their extracurricular time, the students could independently study micro-lectures, watch demonstration videos, complete the online exercises, and practice the screen-based simulation of clinical skills. Students could study difficult problems repeatedly and discuss them with classmates or teachers in the online platform.
3. ***Face-to-face learning in class:*** After a self-study period, students participated in face-to-face learning in class. Teachers administered a five-minute quiz to determine the impact of students’ independent study. According to the results of the in-class quiz, teachers answered questions and guided the students’ thinking and discussion. In contrast to the control group, the teachers in the experimental group abandoned the lecture-based learning model and had more class time for leading discussion and delivering quizzes rather than repeating rote didactics. During skill practice, students voluntarily performed clinical techniques. Students were able to critique other students’ performance, and teachers summarized key practice points. In addition, teachers provided clinical case examples based on real practice, which was used to guide the students’ understanding of the indications, contraindications, operative procedures, and other applications of clinical skill. Unlike traditional teaching methods, the training of theoretical knowledge and skill practice was student-oriented, which motivated students to participate on their own initiative.
4. ***Simulative clinical training:*** Simulative clinical training in the experimental group was conducted in a manner identical to that of the control group. Simulative clinical training included cardiopulmonary resuscitation, tracheal intubation, physical examination of children, neonatal resuscitation, trauma first aid techniques, arterial/venous puncture, aspiration of sputum, gastric tube insertion, dressing change and suture removal, thoracentesis, abdominocentesis, bone marrow puncture, lumbar puncture, pelvic examination, four Leopold maneuvers, and urethral catheterization (Table 2). These skills were the same as those covered in the routine skills practice in internal medicine, surgery, gynecology, pediatrics, emergency care, and nursing. A small class with eight to ten students per group practiced the clinical skills on simulators. Students watched demonstration videos and summarized the key points prior to training. During training, the students repeatedly practiced the clinical skills on simulators and discussed their efforts with each other while teachers provided real-time guidance. After training, the students wrote experimental reports and recorded the operative videos. The teachers reviewed the experimental reports and videos then provided feedback in the next training session.

### Evaluation method

All students involved in the study were tested using the same examinations at the end of the semester in order to evaluate the effects of the different teaching methods used in the experimental and control groups. The test scores of clinical skills included the theoretical and practical components.

#### Examination of theoretical knowledge

We assessed theoretical knowledge using a comprehensive examination of didactic information with a single-choice and multiple-choice test. Ten points were given for each skill. The theoretical examination assessed the indications, contraindications, operative procedures, and other clinical skill applications. Students were given 60 min and 100 points was considered a full score. Computerized software graded the exams using standardized grading.

#### Simulative examination of practice

In order to investigate the students’ comprehensive practical skills, we applied an objective structured clinical examination (OSCE). The simulative examination assessed skills including cardiopulmonary resuscitation, pelvic examination, physical examination of children, lumbar puncture, and urethral catheterization. These skills represent basic techniques used in emergency, gynecology, pediatrics, internal medicine, and surgery. All aspects of the students’ skill practice were evaluated based on preparation, patient evaluation, procedures, post-operative treatments, humanistic care, and medical ethics, according to the scoring standards from the National Medical Practitioner Examination.

#### Student satisfaction

We designed a questionnaire using Sojump (https://www.wjx.cn/) to obtain feedback on the teaching satisfaction from the control and experimental groups. The design of questionnaire was similar to that of Zhou et al. (2020) and covered five main areas: students’ overall satisfaction, content rationality, ease of knowledge acquisition, teacher evaluations, results met expectations. There were five items in the survey. For each item, a four-point scale was provided so that students could rank their responses: 1 (very dissatisfied), 2 (dissatisfied), 3 (satisfied), 4 (very satisfied). Their responses were recorded anonymously in Sojump and the data was analyzed by Cronbach’s *α* test to determine the responses’ internal consistency and reliability.

### Data analysis

All statistical data were analyzed using SPSS (Version 16.0). If data were in gaussian distribution, the data were expressed as mean ±standard deviation and we used a *t-* test to evaluate the two groups. However, if the data showed a non-gaussian distribution, the data were expressed as median/interquartile ranges and non-parametric tests were applied for analysis. A *P* of less than 0.05 was considered to be statistically significant. The effect size was calculated for each result using Cohen’s d calculator to evaluate the magnitude of difference.

### Results

#### Demographic characteristics between the experimental and control groups

The demographic details of all participants in the study were analyzed (Table 1) and the gender, age, and examination scores of the students in their third year of medical college were found to be in a gaussian distribution. The *t-* test was used for the two groups. The age of participants in the control and experimental groups was 21.99 ±0.39 and 22.00 ±0.35, respectively (*t* = 0.192, *p* = 0.848). The examination scores of the control and experimental groups were 84.54 ±4.11 and 85.57 ±3.73, respectively (*t* = 1.864, *p* = 0.064). There were no notable differences in demographic details between the control and experimental groups.

#### Comparing theoretical and practical examination results between the experimental and control groups

We compared the results of the theoretical and practical examinations between the control and experimental groups to evaluate the students’ mastery of clinical skills. Theoretical examination, urethral catheterization, and lumbar puncture data were in accordance with gaussian distribution so the *t*-test was used for analysis. Cardiopulmonary resuscitation, pelvic examination, and physical examination of children data were in a non-gaussian distribution so the Mann–Whitney U was used for analysis. Theoretical examination results from the experimental group were significantly higher than that in the control group, and there was a statistically significant difference between the two groups (*t* = 4.435, *p* < 0.001) (Table 3). A greater effect size was seen for the theoretical scores (Cohen’s *d* = 0.627).

**Table 3:** Comparison of theoretical and practical examination results between the two groups.

Group	Theoretical assessment	Practical assessment				
		CPR	Pelvic examination	Physical examination of children	Urethral catheterization	Lumbar puncture
Experimental group (*n* = 100)	84.055 ± 10.253	90.00 (84.25,95.00)	84.00 (80.00,87.00)	90.00 (86.00, 94.00)	69.050 ± 7.678	86.440 ± 9.982
Control group (*n* = 100)	77.850 ± 9.521	85.00 (76.13,90.00)	80.00 (73.25,82.75)	83.50 (77.25, 90.00)	65.350 ± 6.964	82.010 ± 14.123
*t/z*	4.435	−4.627	−5.775	−5.008	3.569	2.561
*p*	<0.001	<0.001	<0.001	<0.001	0.001	0.02
*Cohen’s d*	0.627	0.809	0.882	0.788	0.507	0.384

The two student groups participated in a clinical skills assessment, which included cardiopulmonary resuscitation, pelvic examination, physical examination of children, urethral catheterization, and lumbar puncture, for the OSCE assessment at the end of the semester. The Cronbach’s *α* coefficient for internal consistency of OSCE station was 0.809. Compared with the control group, the students in the experimental group showed a better performance in cardiopulmonary resuscitation (*z* =  − 4.627, *p* <0.001), pelvic examination (*z* =  − 5.775, *p* <0.001), physical examination of children (*z* =  − 5.008, *p* <0.001), urethral catheterization (*t* = 3.569, *p* = 0.001), and lumbar puncture (*t* = 2.561, *p* = 0.02). A medium- or greater-effect size was noted for cardiopulmonary resuscitation (Cohen’s *d* = 0.809), pelvic examination (Cohen’s *d* = 0.882), physical examination of children (Cohen’s *d* = 0.788), and urethral catheterization (Cohen’s *d* = 0.507). A small effect size was noted for the lumbar puncture (Cohen’s *d* = 0.384).

#### Comparing attitudes towards teaching satisfaction between the experimental and control groups

A total of 200 questionnaires were sent out and had a 100% effective recovery rate. The Cronbach’s *α* coefficient for the five items in the questionnaire was 0.81, which implied that the survey had good internal consistency so we used a *t*-test for analysis. Students’ satisfaction with the teaching method in the experimental group was significantly higher compared to the control group and there was a significant difference in overall satisfaction (*t* = 6.487, *p* < 0.001), content rationality (*t* = 4.569, *p* < 0.001), ease of knowledge acquisition (*t* = 6.585, *p* < 0.001), teacher’s evaluation (*t* = 3.935, *p* <0.001), and results met expectations (*t* = 3.917, *p* <0.001) (Table 4). A medium or greater effect size was note for overall satisfaction (Cohen’s *d* = 0.917), content rationality (Cohen’s *d* = 0.645), ease of knowledge acquisition (Cohen’s *d* = 0.932), teacher’s evaluation (Cohen’s *d* = 0.560), and results met expectations (Cohen’s *d* = 0.554).

**Table 4:** Comparison of teaching satisfaction between two groups (Mean ± SD).

Items	Experimental group (*n* = 100)	Control group (*n* = 100)	*t*	*p*	*Cohen’s d*
Overall satisfaction	3.66 ± 0.52	3.15 ± 0.59	6.487	<0.001	0.917
Content rationality	3.47 ± 0.63	3.03 ± 0.73	4.569	<0.001	0.645
Ease of knowledge acquisition	3.52 ± 0.64	2.88 ± 0.73	6.585	<0.001	0.932
Teacher evaluations	3.48 ± 0.67	3.07 ± 0.79	3.935	<0.001	0.560
Results met expectations	3.43 ± 0.71	3.02 ± 0.77	3.917	<0.001	0.554

## Discussion

Clinical medicine requires highly specialized knowledge, training, and continued education (Zhang et al., 2015). The routine courses that medical students are required to take include theoretical knowledge and clinical practice; these courses are key to building medical students’ competency (Laufer et al., 2015). The enrollment of medical students in China has dramatically increased in the last decade, leading to a relative reduction in opportunities to practice clinical skills. Thus, a variety of teaching methods have been introduced to assist medical students in mastering clinical skills. Our study demonstrated that blended learning could provide individualized specialization training in clinical skills in the face of increased enrollment. Blended learning is student-centered and encourages independent, cooperative, and innovative learning (Coyne et al., 2018).

We conducted theoretical and practical assessments of clinical skill in order to verify the authenticity of subjective evaluation. Our results demonstrated that the experimental group’s theoretical and practical performance were significantly better than the control group’s. Differences were statistically significant. Unlike traditional teaching methods, teachers transitioned from being instructors of knowledge to promoters of study, and students became the leaders of the class, which allowed them to practice subjectively. Students’ learning efficiency improved as they used the online platform at their leisure.

We designed a questionnaire to determine learning satisfaction among the blended learning participants and the controls. Questionnaire results showed that blended learning contributed to the convenient acquisition of knowledge, stimulating student-centered learning, and improved clinical practice. Due to limited class time and number of clinical skills required, it was very important for students to have the ability to learn clinical skills in their extracurricular time (Jang & Kim, 2014). Under the traditional LBL mode, students did not preview the material before class and only listened to lectures as they were presented (Johnson, Chuter & Rooney, 2013). This traditional teaching method did not impress the students and was not conductive to improving their interest in learning and practice (Rose et al., 2016). The use of the Internet is convenient for students, improving their interest in mobile learning without being restricted by class times, laboratory arrangements, and teachers. Blended learning circumvented deficiencies in traditional teaching methods, which helped students manage their learning time independently and improved their active participation.

Operation failures lead to medical liability in clinical practice so there are few opportunities for students to practice clinical skills on patients. The cost of traditional simulators is high and repeated operations can cause damage (Kononowicz et al., 2019). However, the screen-based simulation of clinical skills is safe and can be practiced an unlimited number of times. Screen-based simulation provided students with vivid, realistic scenarios and a strong sense of immersion and interaction consistent with actual operations (Johannesson et al., 2013). We have developed more than 20 screen-based clinical skill simulations, including cardiopulmonary resuscitation, gastric intubation, and lumber puncture. This tool simulated clinical scenarios, indications, preparation, operative procedures, and doctor-patient communication to reproduce clinical scenarios as much as possible (Pfandler et al., 2017). The integration of screen-based simulations with the practice on the simulators enabled students to master the procedures of clinical skills. Screen-based simulation rethinks the traditional teaching pattern and improves the quality of learning and students’ enthusiasm.

### Limitations

There were some limitations to our study, which should be further explored and improved upon in future research. First, teachers spent a great amount of time, energy and material resources to prepare teaching resources and build the online platforms. From the teachers’ perspective, the blended learning of clinical skills posed a new challenge to their knowledge and teaching ability. Teachers should closely follow their syllabus to design and optimize their resources to homogenize teaching. Second, it was recommended that teachers increase students’ participation in class. In the teaching process, we found that student performance was polarized in class. Some students were very active in answering questions and discussion, while some students did not speak or demonstrate at all. In light of this, teachers may need to ask students to demonstrate their opinions and perform the clinical skills in turn to encourage the students’ subjective initiative. Third, all of the blended learning participants in our study were volunteers. Although the analysis of demographic characteristics in both groups showed no difference in age, sex, and examination scores, the participants in the blended learning group may have had a higher active learning ability and were more highly motivated, which influenced the success of that approach. Therefore, future research should randomize participants to prevent potential biases and generate a more robust comparison between the blended learning approach and the traditional teaching method (Cheng et al., 2017). Additionally, the time spent on clinical skills curriculum was identical for the theoretical session and experimental practical sessions in blended learning participants and non-participants. Nevertheless, with regard to the extracurricular time spent on independent learning, students in the traditional teaching method were not required to study the online curricular elements by themselves. Blended learning participants may have spent more time on their studies. Literature has purported that independent learning during extracurricular time is an important part of blended learning and the effects of the blended learning approach cannot be distinguished from the extracurricular time students used. Thus, future studies should consider total time spent on tasks and processing of teaching formats for both blended learning participants and non-participants. Lastly, this pilot study found that students’ practical performance was improved using the blended learning format, but that these achievements were only tracked over one semester. The long-term efficacy of the blended learning approach remains unknown. Therefore, assessing the long-term effects of skills practice should be evaluated in the future.

## Conclusions

We explored the benefits of blended learning on clinical skills compared to a traditional teaching mode. Blended learning used a student-oriented approach for cultivating the clinical practice of competencies for medical students. Applying technologically advanced techniques, such as the screen-based simulation of clinical skills and use of the Internet, was found to increase learning enthusiasm, promote active learning, and improve clinical practice. The application of blended learning may resolve deficiencies in clinical skills practice, compensate for time and space limitations, and ensure teaching efficiency and quality.

## Supplemental Information

Supplemental Information 1 The scores of theoretical and practical examination in clinical skills

Supplemental Information 2 Questionnaire for satisfaction survey

Supplemental Information 3 Original questionnaire

Supplemental Information 4 Questionnaire data

### Additional Information and Declarations

#### Data Availability

The following information was supplied regarding data availability: The scores of theoretical and practical examination in clinical skills are available in the Supplemental Files.
