# Supplementary material for: Application of blended learning approach in clinical skills to stimulate active learning attitudes and improve clinical practice among medical students
Source: PeerJ. 2021 Jun 24;9:e11690. doi: 10.7717/peerj.11690 (PMC8236236; doi:10.7717/peerj.11690)
Supplement: Supplemental Information 2 [file peerj-09-11690-s002.docx]

**Qestionnaire for the teaching activity in clinical skills**

1. I was satisfied with the teaching mode in this course.

□ very dissatisfied □ dissatisfied □ satisfied □ very satisfied

2. The design and content of the teaching mode was rational.

□ very dissatisfied □ dissatisfied □ satisfied □ very satisfied

3. It was easier to acquire knowledge.

□ very dissatisfied □ dissatisfied □ satisfied □ very satisfied

4. Teachers had better teaching effects to stimulate student-center learning.

□ very dissatisfied □ dissatisfied □ satisfied □ very satisfied

5. The results met expectations to improve clinical practice.

□ very dissatisfied □ dissatisfied □ satisfied □ very satisfied
