# Supplementary material for: Application of blended learning approach in clinical skills to stimulate active learning attitudes and improve clinical practice among medical students
Source: PeerJ. 2021 Jun 24;9:e11690. doi: 10.7717/peerj.11690 (PMC8236236; doi:10.7717/peerj.11690)
Supplement: Supplemental Information 3 [file peerj-09-11690-s003.docx]

**问卷调查：临床技能教学满意度调查**

1.我对这门课的教学模式很满意

□非常满意 □满意 □不满意 □非常不满意

1. 教学模式的设计和内容合理

□非常满意 □满意 □不满意 □非常不满意

3.获得知识更容易

□非常满意 □满意 □不满意 □非常不满意

4.教师有较好的教学效果，激发以学生为中心的学习方式

□非常满意 □满意 □不满意 □非常不满意

5.研究的成效达到预期，能改善学生的临床实践能力

□非常满意 □满意 □不满意 □非常不满意
